# Case of a Suppression of Urine, Which Terminated Fatally; with an Account of the Appearances on Dissection

**Published:** 1788

**Authors:** James Stevenson

**Affiliations:** Surgeon at Egham, in Surry


IV. *Case of a suppression of urine, which terminated fatally; with an account of the appearances on dissection. Communicated in a letter to Dr.* Simmons, *by Mr.* James Stevenson, *Surgeon, at Egham, in Surry.*

MR. Stephen Boult, coach-maker, at Staines, in Middlesex, aged sixty-three years, and of a robust habit of body, was seized on Friday, November the 25th, 1785, with violent forcing pains at the bottom of his belly, and an incessant desire to make water, tho' unable to void a single drop. These symptoms were accompanied with a hard throbbing pulse, beating an hundred and twenty strokes in a minute, and with great tension over the whole abdomen.

He

He was extremely thirſty, and his tongue was very dry; he complained alſo of ſickneſs, but did not vomit.

After taking away twenty-four ounces of blood, an oily emollient clyſter was directed to be adminiſtered every four hours, and four table ſpoonfuls of a purgative mixture compoſed of ſoluble tartar, manna, and infuſion of ſena, to be taken every two hours; recourſe was likewiſe had to warm bathing.

On the morning of the 26th his pain was ſomewhat diminiſhed, altho' he had had no ſtool nor any diſcharge of urine. The catheter was now introduced, but without effect, the bladder ſeeming to be quite flaccid.

His pulſe being ſtill very ſtrong, twelve ounces more of blood were directed to be taken away, and the purging medicine, clyſters, and warm bath, were repeated.

Dr. Lind, phyſician at Windſor, who viſited the patient this day, in the evening, recommended a perſeverance in the plan already adopted, with the addition only of half a drachm of diuretic ſalt, to be given every ſix hours in four table ſpoonfuls of a common ſaline mixture.

On the 27th his pulſe was at ninety, and he had had two ſtools, but had made no water; the catheter was therefore again introduced, but with no better ſucceſs than before.

On the 28th the heat of his ſkin was much increaſed, and his pulſe fluctuated between an hundred and an hundred and twenty ſtrokes in a minute. In conſequence of his walking on the cold floor, about an ounce of urine, of a natural colour, was diſcharged drop by drop. During this and the preceding day the purgative and diuretic medicines were continued, with the occaſional uſe of oily clyſters and the warm bath; and in the evening, as the pain in the lower part of the belly was much increaſed, ſix leeches were applied, which bled profuſely.

On the 29th the pain was much relieved. He had had three purgative ſtools, and a little urine had drivelled on the ſheets; but ſtill the tenſion of the belly continued.

On the 30th he had nine ſtools of a dark colour, but voided no urine. His belly was now painful to the touch; his pulſe was much increaſed in quickneſs, and he was ſlightly delirious.

December 1ſt, he had doſed a little in the night, but he now voided his ſtools involuntarily, and was feized with fubfultus tendinum, hiccough and delirium. In this ftate he continued till his death, which happened on the 4th of December, in the morning.

The body was opened about eight hours after death. The inteftines were found perfectly free of difeafe, excepting one blue fpot with a deep red tinge on the curvature of the duodenum. The ftate of the kidneys, ureters, bladder, and all the parts contiguous, was very particularly attended to; but not the leaft appearance of difeafe could be difcovered. To our great furprize, however, more than a pint of pale, inodorous urine was found in the bladder, which appeared to have been very recently fecreted from the kidneys; the catheter having been introduced the night before the patient's death, without the defired effect.

The ftomach was next examined.—The mucus on many parts of it was intirely gone, many fpots were obferved of a deep red, inclining to a blue colour, and the tip of the finger made holes through its fubftance with gentle preffure.

The gall-bladder and ducts had a natural appearance, and altho' two ftones of a large fize were found in the former, there was not the fmalleft inflammation perceptible on either of thefe these parts. From all these circumstances, therefore, we concluded, that the original symptoms of Mr. Boult's disease could not be attributed to any other cause than the local affection of the stomach.

During the progress of this illness, the cause was considered as obscure, but it was agreed by all, that the antiphlogistic plan ought to be pursued, and so it was, very steadily, until symptoms of debility made it manifestly improper.

In short, this disease put on such appearances as left us quite in the dark as to the primary mischief. It militated with the characters commonly laid down of a local affection of the stomach, kidneys, or their appendages.

The true cause could only, I presume, be discovered by the inspection of the body after death, and it most obviously is proved, that it was an idiopathic inflammation of the stomach.

It turned out upon more enquiry that the patient had drank a glass of a very acrid composition * the morning he was taken ill; and it is scarcely to be doubted that this was actually

* This mixture was composed of horseradish, mustard seed, garlic, rue, marshmallow, pimpernel, aniseed, and rhubarb, distilled twice in brandy. I was not able to ascertain the quantities of the several ingredients; but in the distilled liquor actually the immediate cauſe of his death. If this conjecture is well founded, I preſume the caſe will be allowed to differ in ſome of its circumſtances from any upon record, as the inability to make water was immediate and permanent. Is it not probable, that the excretory ducts of the kidneys ſuffered a ſympathetic conſtriction from the ſtimulus applied to the ſtomach?---Is it not likewiſe remarkable, that the patient ſhould never have complained of any pointed, acute pain in the region of the ſtomach; and that he had no vomiting of the ingeſta?---It is true he complained of ſickneſs, yet never vomited; and the pain was confined to the hypogaſtric region, and chiefly to the part juſt above the pubis.—I never heard of ſimilar effects from any fatal poiſon, whoſe operation was immediately upon the coats of the ſtomach, without its producing acute local pain, and violent retching, a burning heat in the epigaſtrium, a rejection of the ingeſta, and quor, which was ſo uncommonly acrid and pungent that I ſhould have thought it hardly poſſible for any perſon to ſwallow an ounce of it, the taſte of the horſeradiſh was extremely predominant. This poor man, who often tried his ſkill upon others to cure the evil, rheumatiſm, and other diſeaſes, had diſtilled between thirty and forty gallons of this liquor, for the purpoſe of curing his friends, but fortunately for them took the firſt fatal doſe himſelf, for a rheumatic complaint, to which he had been frequently ſubject.

commonly, I believe, hiccough. All thefe fymptoms this patient was free from, and yet manifeft inflammation, in an extenfive degree, was evident upon infpection of the body.

Laurel water, as is well known, deftroys animals, but without producing any evident local mifchief in the ftomach, its operation feeming to be entirely on the nervous fyftem. The Fox Glove feems to have a power of the fame kind, and fo perhaps have many other vegetable poifons; but I am acquainted with none whofe effects exactly refemble thofe of the compofition which proved fatal in the prefent inftance.

Altho' I had no doubt, after the difcovery of this poifon, that it had been the caufe of Mr. Boult's death, I procured fome of it, and gave two ounces to a dog of a middle fize, about ten o'clock in the morning. The dog, after fwallowing this dofe, ran about for the fpace of an hour, and then flept for about two hours, when he waked apparently in great pain, refufed both victuals and drink, and was convulfed. About twelve o'clock at night, I was inclined to put an end to his mifery, but for the fake of the experiment it was determined to wait the final effect of the poifon, which probably took place foon after, after, as he was found cold and ſtiff at five o'clock the next morning.

I opened the dog, and the appearances were nearly the ſame as thoſe obſerved in Mr. Boult's ſtomach. The only difference in the two caſes was in the diſtance of time from the adminiſtering the poiſon to the death. The dog never vomited altho' he ſwallowed more than Mr. Boult, for the glaſs the latter took it from could not hold quite two ounces.

